# Recent Floods

**Published:** 1890-06

**Authors:** 


### RECENT FLOODS.

If any person is in a position to observe the ravages of river floods, an inhabitant of Cairo, Ill., might reasonably be supposed to have unusual facilities for it, and a late article in the *Cleveland Leader*, by C. R. Woodward, of the first named city, upon this subject, would appear to justify our conclusion.

Mr. Woodward suggests as a remedy, the establishment of a series of reservoirs, or back-sets at the mouths of tributary streams, serving also as aids to navigation by increasing the water supply in the dry season. Lake Winnepesaukee, in New Hampshire, has been made available in this manner, for increasing the supply of water in the Merrimac, to turn the mill wheels of Manchester and Lowell, in times of low water.

The woful destruction of life and property, and impairment of health along the course of the Ohio and Mississippi rivers alone, make it imperative that something should be done to avert or at least lessen the recurrence of like calamities.

